# Musculoskeletal Complaint Patterns and Training Impact in Adult Triathletes During the 2023 Season

**DOI:** 10.3390/jfmk11040402

**Published:** 2026-10-03

**Authors:** Jeffrey J. Parr, Susan R. Dobson, Jonathan Navara, Mounica Kusuma, Sahil Hareshbhai Kothiya, Felix Twum

**Affiliations:** School of Health Professions, University of Southern Mississippi, Hattiesburg, MS 39406, USA; susan.dobson@usm.edu (S.R.D.); felix.twum@usm.edu (F.T.)

**Keywords:** triathlon, musculoskeletal complaints, overuse complaints, running, endurance athletes, sports epidemiology, injury surveillance

## Abstract

**Objectives**: To characterize self-report musculoskeletal complaints, anatomical distribution, healthcare utilization, training impact, and associations with demographic and training characteristics among adult triathletes during the 2023 season. **Methods**: A retrospective cross-sectional survey was conducted among community-based triathletes recruited through social media and triathlon organizations. Adults aged ≥18 years who completed or intended to complete at least one triathlon in 2023 were included (*N* = 245). Demographic characteristics, years of participation, race focus, and weekly training volume were collected. Main outcomes included self-reported musculoskeletal complaints, anatomical location, complaint classification, healthcare utilization, and training disruption. **Results**: Of 245 participants (60.0% male; most frequent age group 30–34 years), 127 of 242 with available complaint data (52.5%) reported at least one complaint, totaling 175 reported complaints. The lower extremity accounted for 79.4% of complaints, most commonly the hip/pelvis (22.3%), lower leg (18.7%), knee (17.3%), ankle (15.1%), and foot/toes (15.1%). Running was the most frequently reported activity associated with complaint onset (61%). The number of reported complaints was not significantly associated with the broad demographic or training characteristics examined. Healthcare utilization was reported for 62.4% of complaints with available healthcare data. Among complaints with available training impact data, 50.6% resulted in both missed and modified training, 28.2% in modified training only, and 21.2% in missed training only. **Conclusions**: More than half of the triathletes surveyed reported musculoskeletal complaints during the 2023 season, with lower-extremity and overuse complaints predominating and running most frequently associated with complaint onset. Broad demographic and general training characteristics were not significantly associated with the number of reported complaints. These findings highlight the burden and training consequences of self-reported musculoskeletal complaints among adult triathletes and support the need for prospective surveillance incorporating discipline-specific exposure and more detailed training load measures.

## 1. Introduction

Triathlon comprises three distinct sports disciplines: swimming, cycling, and running, and has recently gained global recognition as a relatively young sport [1,2]. Triathletes train on average 10–14 h a week, comprising 8–10 training sessions, which is higher than single-sport athletes, thus increasing the risk of musculoskeletal complaints [3,4]. These demands expose athletes to repetitive loading across multiple movement patterns and may contribute to musculoskeletal complaints that interfere with training and competition. Understanding the frequency, characteristics, and functional consequences of these complaints is therefore important for clinicians and coaches involved in triathlon injury prevention and management.

Musculoskeletal complaints are common among triathletes, although estimates vary considerably according to the population studied, injury definition, surveillance period, and method of data collection. Recent prospective surveillance of elite short-course triathletes reported substantial injury incidence and prevalence [5], while a recent systematic review similarly identified overuse and lower-extremity conditions as prominent health problems in short-course triathletes [6]. Studies of recreational and long-distance triathletes have likewise reported a predominance of overuse complaints and lower-extremity involvement [7,8]. Running is frequently identified as the discipline associated with the greatest number of reported musculoskeletal complaints, with the knee, lower leg, ankle, and foot commonly affected [5,6,7,9,10,11,12,13,14,15,16].

Despite this literature, important gaps remain. Contemporary studies have often focused on elite athletes, specific race distances, individual events, or geographically defined populations, while methodological differences in injury definitions and surveillance approaches limit comparisons across studies. Recent work has strengthened understanding of injury occurrence in elite short-course triathletes [5,6] but less contemporary information is available across the broader adult triathlon population. Moreover, prevalence and anatomical distribution alone do not fully characterize the clinical impact of musculoskeletal complaints. Information regarding whether athletes seek healthcare or modify or miss training provides additional insight into the functional consequences of these complaints and their relevance to sports medicine and rehabilitation.

Our previous investigation characterized injury patterns and perceived preventive strategies among triathletes during the 2022 season [17]. The present study was designed as an independent assessment of musculoskeletal complaint patterns during the subsequent 2023 season, with additional emphasis on anatomical distribution, healthcare utilization, and training disruption. Accordingly, the primary purpose of this study was to characterize self-reported musculoskeletal complaints among adult triathletes during the 2023 season, including their anatomical distribution, classification, associated triathlon discipline, healthcare utilization, and impact on training. A secondary purpose was to examine associations between selected demographic and general training characteristics and the number of reported musculoskeletal complaints. We hypothesized that lower-extremity and overuse complaints would predominate, that running would be the discipline most frequently associated with reported complaints, and that greater training volume would be associated with a greater number of reported musculoskeletal complaints.

## 2. Materials and Methods

### 2.1. Study Design

A retrospective cross-sectional online survey was used to characterize self-reported musculoskeletal complaints, triathlon participation and training characteristics, healthcare utilization, and training disruption among adult triathletes during the 2023 calendar year. Data were collected using Qualtrics (Qualtrics, Provo, UT, USA). The survey was distributed beginning 1 January 2024 and remained open for 75 days. Recruitment occurred through triathlon-specific social media groups, triathlon clubs, and email distribution to large triathlon organizations and international federations.

### 2.2. Ethical Considerations

The study was conducted in accordance with the Declaration of Helsinki and was approved by the Institutional Review Board of The University of Southern Mississippi (protocol 23-0950; approved 8 December 2023). All participants provided informed consent electronically before completing the survey.

### 2.3. Participants

Individuals were eligible to enter the survey if they were aged ≥18 years, could read and understand English, and had raced or intended to race at least one triathlon during the 2023 calendar year. Recruitment used an open convenience-sampling approach through triathlon-specific social media groups, clubs, and organizational mailing lists. Because the total number of individuals exposed to the survey invitation and the size of the accessible source population could not be determined, neither a formal response rate nor a population-based sample-size calculation was available. Recruitment remained open for the prespecified 75-day collection period, and all eligible responses received during that period were included.

A total of 282 individuals consented to participate. Following eligibility and data-quality screening, 245 respondents were retained for analysis. Recruitment used convenience sampling through triathlon-specific social media groups, clubs, and organizational mailing lists. Because distribution links could be viewed or redistributed through these channels, the total number of individuals exposed to the survey invitation could not be determined, and a formal response rate could not be calculated. Consequently, the sample should not be interpreted as a probability-based or nationally representative sample of triathletes.

### 2.4. Procedures

The survey was adapted from the instrument used in our previous investigation of triathletes during the 2022 season [17]. The original instrument was developed by sports medicine researchers with experience in triathlon participation and injury research and was pilot tested with local triathletes to improve question clarity and survey usability. The survey used in the present study was subsequently reviewed by three sports medicine researchers before deployment. Formal psychometric testing, including test–retest reliability, construct validity, and criterion validity, was not performed.

Demographic variables collected to characterize the study sample included age group, gender, race/ethnicity, geographical region, marital status, educational attainment, and annual household income. Triathlon-specific variables included race category (age group, elite, physically challenged, or collegiate), years of triathlon participation, number of races started by race distance, primary race-distance focus, average weekly training hours, training emphasis, number of races not completed (did-not-finish [DNF]), reasons for DNF, and whether a participant did not start a registered race because of a musculoskeletal complaint. Variables selected for inferential analyses based on the study objectives included age, gender, race category, primary race-distance focus, years of triathlon participation, and weekly training hours.

Participants could report up to four musculoskeletal complaints experienced during the 2023 calendar year. For the purposes of the survey, a musculoskeletal complaint was defined as any physical condition causing pain during training or racing. This symptom-based definition was intentionally broad to capture conditions affecting training that may not have resulted in time loss, medical attention, or a formal clinical diagnosis. Accordingly, the outcomes in the present study are interpreted as self-reported musculoskeletal complaints rather than medically verified injuries.

For each reported complaint, participants identified the general and specific anatomical location and indicated whether they sought care from a healthcare professional; whether they considered the complaint acute or overuse; the activity most closely associated with the complaint (swimming, cycling, running, strength training, or unsure); and whether the complaint resulted in missed training, modified training, or both. Participants reporting missed or modified training selected the duration as 1–3, 4–7, 8–14, 15–28, or ≥29 days.

Anatomical locations were categorized using a modified framework aligned with Orchard Sports Injury and Illness Classification System regions. Two researchers reviewed responses for completeness and internal consistency, and four researchers trained in biostatistics and epidemiology independently coded the data. Coding discrepancies were resolved through consensus.

### 2.5. Musculoskeletal Complaint Definition

The primary outcome was a self-reported musculoskeletal complaint occurring during triathlon training or racing. A complaint was defined as “any physical condition causing pain during training or racing.” This definition was selected to capture the broader spectrum of training-related health problems, including complaints that may alter participation without resulting in complete time loss or formal medical attention. This approach is conceptually consistent with contemporary surveillance frameworks that recognize that time-loss definitions alone may underestimate the burden of health problems in athletes [18,19]. Because complaints were self-reported and were not independently confirmed by a healthcare professional, the present study does not interpret these events as medically diagnosed injuries.

### 2.6. Data Quality and Bias

Survey responses were reviewed for eligibility, completeness, and internal consistency before analysis. However, because recruitment occurred through open online channels, participant identity, federation membership, and race participation could not be independently verified. The use of voluntary convenience sampling also introduces the potential for selection and non-response bias; individuals experiencing musculoskeletal complaints may have been more motivated to participate than asymptomatic triathletes. In addition, retrospective reporting across a calendar year introduces the possibility of recall and classification bias. These limitations were considered when interpreting estimates from the sample and restrict generalization to the broader triathlon population.

### 2.7. Statistical Analyses

All statistical analyses were conducted using IBM SPSS Statistics (version 31.0; IBM Corp., Armonk, NY, USA) and R statistical software (version 4.3.3; R Foundation for Statistical Computing, Vienna, Austria). Descriptive statistics were calculated for all demographic, training, and musculoskeletal complaint variables. Frequencies and corresponding 95% confidence intervals (CI) were calculated for musculoskeletal complaint outcomes.

Associations between age, gender, race category, primary race-distance focus, years of triathlon participation, weekly training hours, and musculoskeletal complaint occurrence were examined using chi-square tests, with Fisher’s exact tests used when expected cell counts did not meet chi-square assumptions. Associations were examined between demographic and training variables (age, gender, race category, race focus, years of triathlon participation, and weekly training hours) and complaint occurrence.

To further examine factors associated with the number of complaints reported (count outcome), a discrete Weibull regression model was used due to its flexibility in modeling potentially dispersed count data. This approach allows for appropriate modeling of count outcomes that may not meet the assumptions of traditional Poisson regression. Model coefficients are presented with corresponding 95% confidence intervals and *p*-values.

Exposure-adjusted injury rates were not calculated because athlete-specific exposure data (e.g., discipline-specific training volume or duration) were not available. Cases with missing categorical data (<5%) were excluded using listwise deletion. Statistical significance was set a priori at *p* < 0.05.

## 3. Results

Of the 282 individuals who consented to participate, 245 (86.9%) provided complete or partial responses to the musculoskeletal complaint items and were included in the analytic sample, while 37 participants (13.1%) were excluded due to incomplete musculoskeletal complaint data or ineligibility (e.g., no triathlon participation during the 2023 season). The sample was predominantly male (*n* = 145, 60.0%), White (*n* = 213, 88.0%), and most frequently in the 30–34-year age category. Age and gender distribution can be seen in Table 1.

Most participants resided in the United States (*n* = 199, 82.0%), representing 38 states and the District of Columbia. Nearly all participants who reported a race category competed as age-group athletes (*n* = 234, 95.5%), with smaller proportions competing in collegiate (*n* = 8, 3.3%) and professional/elite (*n* = 3, 1.2%) categories. Among participants reporting a primary race-distance focus, half-Iron/70.3 was most common (*n* = 72, 29.5%), followed by sprint (*n* = 69, 28.3%), full-Iron/140.6 (*n* = 61, 25.0%), and Olympic distance (*n* = 42, 17.2%). Participants reported a mean of 8.17 ± 8.21 years of triathlon experience (range, 0–42 years). Additional sociodemographic and training characteristics can be found in Table 2. A slight majority described their training approach as balanced (51.3%), and the largest proportion trained 6 to 10 h per week (41.7%). Twenty participants (8.2%) reported at least one did-not-finish (DNF), of whom eight (40.0%) attributed the DNF to injury. Additionally, 33 participants (13.6%) reported at least one did-not-start (DNS) for a registered race due to injury.

During the 2023 season, 127 participants (52.5%; 95% CI, 46.0–58.9%) self-reported at least one musculoskeletal complaint. Among participants reporting a complaint, 85 (67.0%) reported one complaint, 36 (28.3%) reported two complaints, and 6 (4.7%) reported three complaints, for a total of 175 reported complaints. The number of complaints reported per participant was not significantly associated with age, gender, race category, primary race-distance focus, years of triathlon participation, or average weekly training hours (Table 3).

**Table 3:** Associations Between Participant Characteristics and Number of Self-Reported Complaints.

Variables	Statistic	df	* *p*-Value
Age vs. # of Complaints	34.58	36	0.54
Gender vs. # of Complaints	0.51	3	0.92
Race Category vs. # of Complaints	5.25	6	0.51
Focus Race vs. # of Complaints	8.17	9	0.52
Triathlon Years vs. # of Complaints	81.54	96	0.85
Training Hours vs. # of Complaints	12.67	12	0.39

* Discrete Weibull regression identified no statistically significant associations between the demographic or general training characteristics evaluated and the number of reported musculoskeletal complaints (Table S1). The overall model did not significantly improve fit compared with the intercept-only model (likelihood-ratio χ^2^(9) = 3.76, *p* = 0.927). The lower extremity accounted for the majority of reported musculoskeletal complaints (79.4%; 95% CI, 72.7–85.2%), with the hip/pelvis representing the most frequently reported lower-extremity location. A detailed anatomical distribution is presented in Table 4. # = number.

**Table 4:** Anatomical Distribution of Self-Reported Musculoskeletal Complaints.

Anatomical Site	*N* (% *)	*N* (%)
Lower Extremity		139 (79.4%)
Ankle	21 (15.1%)	
Foot/Toes	21 (15.1%)	
Hip/Pelvis	31 (22.3%)	
Knee	24 (17.3%)	
Lower Leg	26 (18.7%)	
Thigh	14 (10.1%)	
Other/Unspecified	2 (1.4%)	
Upper Extremity		16 (9.1%)
Elbow	2 (12.5%)	
Hand/Fingers	3 (18.8%)	
Shoulder/Clavicle	10 (62.5%)	
Wrist	1 (6.3%)	
Spine		10 (5.7%)
Cervical (Neck)	0 (0%)	
Thoracic (Mid-Back)	0 (0%)	
Lumbar (Low Back)	10 (100%)	
Torso		6 (3.4%)
Abdomen	1 (16.7%)	
Ribs	2 (33.3%)	
Other organs	3 (50.0%)	
Head/Face		4 (2.3%)
Brain/Concussion	3 (75%)	
Teeth	1 (25%)	
Total	175	

* Percentage calculated using the total number of complaints within the corresponding general anatomical region as the denominator.

Of the 175 self-reported musculoskeletal complaints, healthcare utilization (e.g., physician, physical therapist, certified athletic trainer) was reported for 173 complaints. Of these, 108 (62.4%) resulted in healthcare utilization (95% CI, 55.0–69.3%). Activity associated with complaint onset was reported for 172 complaints. Running was the most frequently identified activity (*n* = 105, 61.0%; 95% CI 53.6–68.0), followed by cycling (*n* = 17, 9.9%), swimming (*n* = 9, 5.2%), and strength training (*n* = 6, 3.5%); for 35 complaints (20.3%), participants were unsure of the associated activity. Training impact data were available for 170 complaints. Of these, 86 (50.6%) resulted in both training modification and missed training, 48 (28.2%) resulted in training modification without missed training, and 36 (21.2%) resulted in missed training without modification.

Weekly training hours differed significantly by gender and primary race-distance focus but were not significantly associated with age, race category, or years of triathlon participation (Table S2).

**Figure 1:**
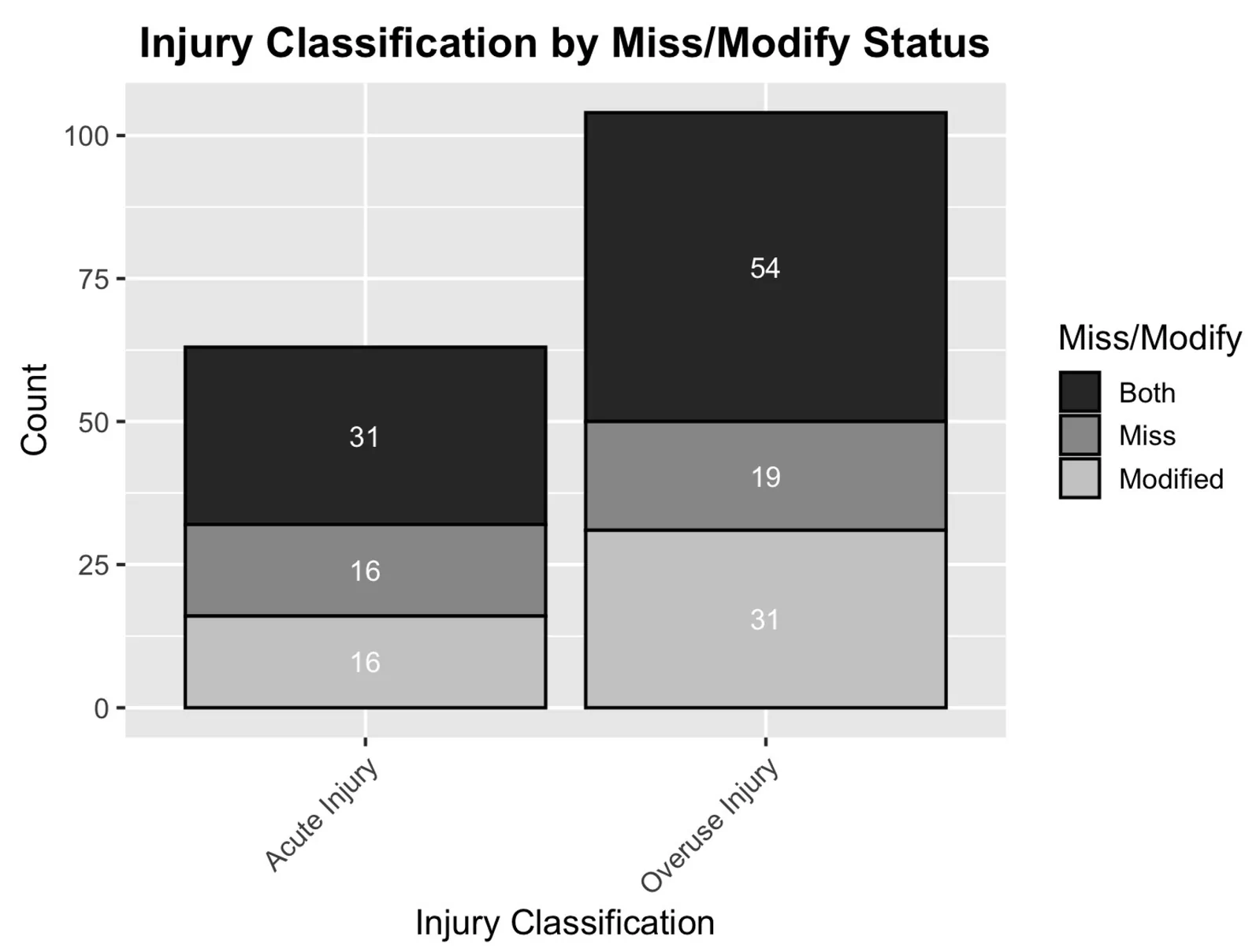
Distribution of self-reported musculoskeletal complaints classified as acute or overuse, stratified by whether complaints resulted in missed training time, modified training, or both.

Acute-versus-overuse classification was available for 170 of the 175 self-reported musculoskeletal complaints. Of these, 106 (62.4%) were classified by participants as overuse and 64 (37.6%) as acute. Complaint classification (acute versus overuse) was not significantly associated with training impact (missed training, modified training, or both), χ^2^(2, *N* = 167) = 1.28, *p* = 0.53, Cramer’s V = 0.09 (Figure 1).

## 4. Discussion

### 4.1. Main Findings

This study characterized self-reported musculoskeletal complaints, healthcare utilization, and training impact among adult triathletes during the 2023 season. More than half of the participants reported at least one complaint, consistent with the substantial burden of injury and musculoskeletal complaints reported in previous triathlon research [7,10,17,20,21]. The lower extremity accounted for nearly four-fifths of reported complaints, and running was the activity most frequently associated with complaint onset among complaints for which an activity was identified. These findings are broadly consistent with previous research identifying running and lower-extremity conditions as prominent sources of injury burden among triathletes [5,6,7,13,16]. However, because discipline-specific training exposure was not measured, the greater number of complaints associated with running should not be interpreted as evidence that running carried greater injury risk than cycling or swimming. More than 60% of reported complaints prompted participants to seek healthcare, while complaints with available training impact data were characterized by missed training, modified training, or both, demonstrating that many of the reported complaints had meaningful consequences for training participation within this sample.

The anatomical distribution of complaints was dominated by the lower extremity, with hip/pelvis representing the largest proportion, followed by the lower leg, knee, ankle, and foot. The prominence of hip/pelvic complaints differs from previous triathlon literature, in which the knee and spine have commonly been identified among the most frequently affected anatomical regions [13]. This finding should be interpreted cautiously because the present study did not collect diagnostic information or biomechanical measures that would identify the specific structures or mechanisms underlying hip/pelvic complaints. Running-related injury research suggests that proximal lower-extremity biomechanics may contribute to injury development in some populations; however, systematic reviews have found that associations between individual biomechanical characteristics and running-related injury are generally inconsistent [22]. Therefore, the relatively high proportion of hip/pelvic complaints observed in this sample should be considered hypothesis-generating rather than evidence of a distinct injury mechanism or change in triathlon injury patterns. Future prospective studies incorporating diagnostic classification, discipline-specific training exposure, and biomechanical assessment may help determine whether this finding reflects differences in training load, athlete characteristics, or other factors.

Overuse complaints predominated in the present study, accounting for 62.4% of complaints with available classification data, compared with 37.6% classified as acute. This distribution is consistent with previous triathlon research demonstrating a predominance of overuse conditions, particularly among long-distance triathletes [13]. Repetitive loading associated with endurance training provides a plausible context for the high proportion of overuse complaints; however, the present study did not collect discipline-specific exposure or longitudinal training load data necessary to examine load-related mechanisms. Complaint classification was not significantly associated with training impact, suggesting that both acute and overuse complaints may meaningfully disrupt training. In addition, 62.4% of complaints with available healthcare-utilization data resulted in the participant seeking care from a healthcare provider. Together, the frequency of training disruption and healthcare utilization demonstrates that the self-reported complaints captured in this study were not limited to transient symptoms without consequences for participation or clinical care.

No significant associations were identified between the number of reported complaints and the broad demographic and training characteristics examined in this study, including age, gender, race category, years of triathlon participation, and average weekly training hours. These findings should not be interpreted as evidence that training-related or athlete-specific risk factors are absent. Average weekly training hours provide only a general measure of training exposure and do not account for discipline-specific volume, training intensity, changes in workload over time, or the mechanical loading experienced by individual tissues. Training load research has emphasized that injury risk may depend not simply on the absolute amount of training performed but on how training load is accumulated and progressed relative to an athlete’s capacity [23]. More recent injury models similarly emphasize the interaction among tissue loading, tissue capacity, and accumulated damage, highlighting limitations of broad cumulative training measures for estimating injury risk [24]. Therefore, the absence of associations with weekly training hours in the present study should be interpreted within the limitations of the exposure measures available rather than as evidence that training load is unrelated to musculoskeletal complaints.

These findings have practical implications for clinicians and coaches working with triathletes, although they should not be interpreted as establishing specific injury-prevention interventions. The predominance of lower-extremity complaints and the frequency with which running was associated with complaint onset support careful consideration of running-related symptoms when evaluating and managing triathletes. However, evidence linking specific running training parameters to injury remains inconsistent, and universal recommendations regarding training volume or progression are not currently supported [25]. Similarly, although exercise-based interventions are frequently incorporated into injury-prevention programs, recent evidence does not demonstrate a consistent overall reduction in running-related injuries among endurance runners [26]. Accordingly, prevention strategies for triathletes may be better individualized according to an athlete’s complaint history, training demands, clinical presentation, and tolerance to training rather than based solely on broad demographic characteristics or weekly training volume.

### 4.2. Limitations

This study has several limitations. First, data were collected retrospectively through a self-reported survey, introducing the potential for recall and classification bias. The symptom-based definition used to identify complaints was intentionally broad and did not require medical diagnosis or time loss; consequently, the findings should be interpreted as self-reported musculoskeletal complaints rather than medically verified injuries. Although a substantial proportion of reported complaints resulted in healthcare utilization or disruption of training, the broad definition may have captured conditions of varying clinical severity.

Second, participants were recruited through convenience sampling using social media, triathlon clubs, organizations, and federations. Because the number of athletes exposed to recruitment materials was unknown, a response rate could not be calculated, and selection and nonresponse bias cannot be excluded. Athlete identity, federation membership, and participation in or intention to participate in a 2023 triathlon could not be independently verified. The sample was also predominantly White, highly educated, and composed primarily of age-group triathletes, limiting generalizability to more diverse populations and to elite, collegiate, or youth triathletes.

Third, although the survey was adapted from an instrument used in previous research and reviewed by sports medicine researchers, formal psychometric evaluation was not performed. The cross-sectional design also precludes establishing temporal or causal relationships between participant or training characteristics and complaint occurrence. Furthermore, training exposure was measured only as average weekly training hours. Discipline-specific volume, intensity, changes in training load over time, and exposure within swimming, cycling, and running were not collected. Therefore, exposure-adjusted incidence rates could not be calculated, and the observed frequency of complaints associated with individual disciplines should not be interpreted as relative injury risk. These limitations also restrict conclusions regarding potential training-related risk factors and mechanisms underlying the distribution of complaints observed.

### 4.3. Future Research

Future research should use prospective injury-surveillance designs that incorporate discipline-specific exposure and objective or prospectively recorded training load measures. Quantifying swimming, cycling, and running volume and intensity would allow calculation of exposure-adjusted injury rates and more direct evaluation of relationships between training characteristics and complaint occurrence. Future studies should also consider incorporating data from wearable devices to provide objective measures of training volume and intensity. Such data may allow more detailed examination of discipline-specific training exposure and changes in training load in relation to musculoskeletal complaints. Prospective studies should also incorporate standardized diagnostic classification where feasible to distinguish medically diagnosed injuries from broader musculoskeletal symptoms and to characterize specific conditions affecting the hip/pelvis and other commonly reported anatomical regions.

The relatively high proportion of hip/pelvic complaints observed in this sample warrants further investigation using biomechanical and clinical measures capable of evaluating potential mechanisms rather than relying on anatomical location alone. Future studies may also examine how training load progression, previous injury, recovery characteristics, and athlete-specific factors interact over time to influence injury susceptibility. Finally, surveillance across more diverse triathlon populations, including athletes of different competitive levels and geographic and demographic backgrounds, would help determine the generalizability of the musculoskeletal complaint patterns observed in the present study.

## 5. Conclusions

More than half of surveyed adult triathletes reported at least one musculoskeletal complaint during the 2023 season, with the lower extremity accounting for the majority of reported complaints and running most frequently identified as the activity associated with complaint onset. Hip/pelvic complaints represented the largest proportion of lower-extremity complaints in this sample, differing from some previous triathlon literature and warranting further investigation rather than interpretation as evidence of a distinct epidemiological pattern. Overuse complaints were more common than acute complaints, and complaint classification was not significantly associated with training impact category. In addition, a substantial proportion of reported complaints resulted in healthcare utilization, indicating that many complaints prompted clinical evaluation or treatment.

Broad demographic and training characteristics, including average weekly training hours, were not significantly associated with the number of reported complaints; however, these findings should not be interpreted as evidence that athlete- or training-related risk factors are absent. Prospective surveillance incorporating discipline-specific exposure, objective training load measures, and standardized diagnostic classification is needed to better characterize injury risk and inform evidence-based prevention and clinical management strategies in triathletes.

## Figures and Tables

**Table 1:** Age Distribution of Participants by Gender.

Age Group	Female *N* = 97 *	Male *N* = 145 *	Overall *N* = 242 *
18–19	1 (1.0%)	2 (1.4%)	3 (1.2%)
20–24	7 (7.2%)	2 (1.4%)	9 (3.7%)
25–29	17 (18%)	18 (12%)	35 (14%)
30–34	21 (22%)	28 (19%)	49 (20%)
35–39	11 (11%)	28 (19%)	39 (16%)
40–44	10 (10%)	10 (6.9%)	20 (8.3%)
45–49	5 (5.2%)	14 (9.7%)	19 (7.9%)
50–54	10 (10%)	13 (9.0%)	23 (9.5%)
55–59	7 (7.2%)	9 (6.2%)	16 (6.6%)
60–64	1 (1.0%)	8 (5.5%)	9 (3.7%)
65–69	3 (3.1%)	8 (5.5%)	11 (4.5%)
70–74	4 (4.1%)	4 (2.8%)	8 (3.3%)
75–79	0 (0%)	1 (0.7%)	1 (0.4%)

* *n* (%).

**Table 2:** Marital Status, Education, Annual Income, Race/Ethnicity, Training Hours.

Variable	Number (%)
Marital Status	
Married	151 (62.4%)
Widowed	2 (0.8%)
Divorced	19 (7.9%)
Never Married	64 (26.4%)
Separated	2 (0.8%)
Prefer not to answer	4 (1.7%)
Education	
Less than high school	1 (0.4%)
High school graduate	2 (0.8%)
Some college/currently enrolled	18 (7.4%)
2-year degree	7 (2.9%)
4-year degree/Bachelor’s degree	96 (39.7%)
Master’s degree	76 (31.4%)
Doctorate	32 (13.2%)
Professional degree	10 (4.1%)
Income	
Less than $29,999	10 (4.1%)
$30,000-$59,999	19 (7.9%)
$60,000-$99,999	56 (23.1%)
$100,000 or more	157 (64.9%)
Race/Ethnicity	
White, non-Hispanic	213 (88.0%)
Black or African American	1 (0.4%)
Hispanic	12 (5.0%)
Asian, non-Hispanic	6 (2.5%)
Multiracial	4 (1.7%)
Some Other Race, Non-Hispanic	2 (0.8%)
American Indian or Alaska Native	1 (0.4%)
Prefer not to answer	3 (1.2%)
Training Hours	
0–5 h	16 (6.6%)
6–10 h	101 (41.7%)
11–15 h	89 (36.8%)
16–20 h	30 (12.4%)
20+ h	6 (2.5%)
Total	242

## Data Availability

The raw data supporting the conclusions of this article will be made available by the authors on request.

## Supplementary Materials

The following supporting information can be downloaded at: https://www.mdpi.com/article/10.3390/jfmk11040402/s1, Table S1: Discrete Weibull regression examining associations between participant characteristics and number of self-reported musculoskeletal complaints; Table S2: Associations Between Participant Characteristics and Average Weekly Training Hours.

## Abbreviations

The following abbreviations are used in this manuscript:

DNF	Did Not Finish
DNS	Did Not Start
CI	Confidence Interval
