# Supplementary material for: Musculoskeletal Complaint Patterns and Training Impact in Adult Triathletes During the 2023 Season
Source: J Funct Morphol Kinesiol. 2026 Oct 3;11(4):402. doi: 10.3390/jfmk11040402 (PMC13692321; doi:10.3390/jfmk11040402)
Supplement: Supplementary file 1 [file jfmk-11-00402-s001.zip › jfmk-4596207-supplementary.pdf]

Table S1. Discrete Weibull regression examining associations between participant characteristics and number of self-reported musculoskeletal complaints.

Variable	β	95% CI	p-value
Age (per 5-year category)	0.012	−0.059 to 0.082	0.745
Gender: Female vs. Male	−0.009	−0.303 to 0.285	0.951
Race category: Collegiate vs. Age-group	0.237	−0.622 to 1.095	0.589
Race category: Professional/Elite vs. Age-group	0.728	−0.597 to 2.053	0.282
Primary race distance: Olympic vs. Sprint	−0.222	−0.666 to 0.221	0.325
Primary race distance: Half-Iron/70.3 vs. Sprint	−0.202	−0.603 to 0.200	0.325
Primary race distance: Full-Iron/140.6 vs. Sprint	−0.128	−0.582 to 0.325	0.580
Years of triathlon participation	0.005	−0.018 to 0.028	0.693
Weekly training hours	0.002	−0.041 to 0.044	0.944

Note: β = regression coefficient; CI = confidence interval. Reference categories were Male for gender, Age-group for race category, and Sprint for primary race distance. The analysis included 240 participants with complete data. Overall model fit: log-likelihood = −263.37; Akaike information criterion (AIC) = 548.75; Bayesian information criterion (BIC) = 587.04. Compared with the intercept-only model, inclusion of the predictor variables did not significantly improve model fit, likelihood-ratio $\chi^2(9) = 3.76$, $p = 0.927$.

Table S2. Associations Between Participant Characteristics and Average Weekly Training Hours

Variables	Test	Statistic	df	p-value
Training Hours vs Age	Chi-sq	50.06	48	0.39
Training Hours vs Gender	Fisher			**0.01**
Training Hours vs Race Category	Chi-sq	10.10	8	0.26
Training Hours vs Focus Race	Chi-sq	78.45	12	**0.00**
Training Hours vs Triathlons Years	Chi-sq	138.67	128	0.24
